# Intra-Articular Dislocation of the Patella

**DOI:** 10.1155/2013/535803

**Published:** 2013-02-26

**Authors:** Gavin McHugh, Ed Ryan, May Cleary, Paddy Kenny, Shea O'Flanagan, Peter Keogh

**Affiliations:** Department of Orthopaedic Surgery, Connolly Memorial Hospital, Blanchardstown, Dublin 15, Ireland

## Abstract

We present an unusual case of a chronic, irreducible intra-articular dislocation of the patella in an elderly nursing home resident. The patient had been unable to weight bear for 3 weeks. Radiographs in the emergency department (ED) confirmed the intra-articular dislocation with the superior pole lodged in the intercondylar notch. She underwent two failed closed reduction attempts and subsequently required an open reduction under general anaesthesia.

## 1. Introduction

The vast majority of traumatic dislocations of the patella involve lateral displacement of the patella and are normally reduced either spontaneously or with closed manipulation by simply extending the knee joint. We present the case of an intra-articular dislocation with a failed closed reduction necessitating an open procedure.

## 2. Case History

An 80-year-old lady, resident in a nursing home, presented to the ED with a 3-week history of a painful right knee and inability to mobilise. She had a minor stumble prior to this, but the nursing staff members were unable to confirm any definite trauma.

**Figure 1:**
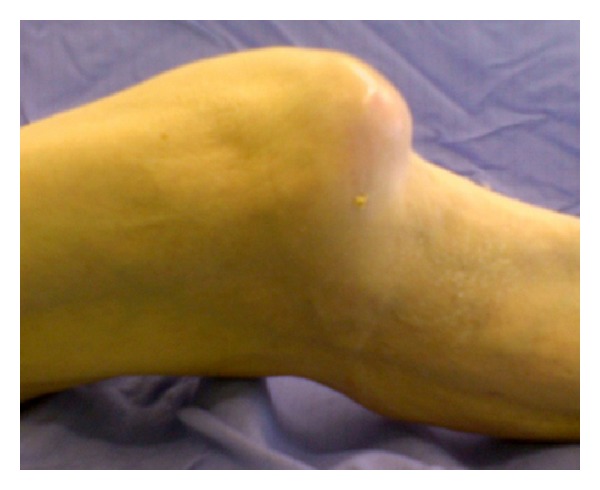
Lateral view of the knee.

**Figure 2:**
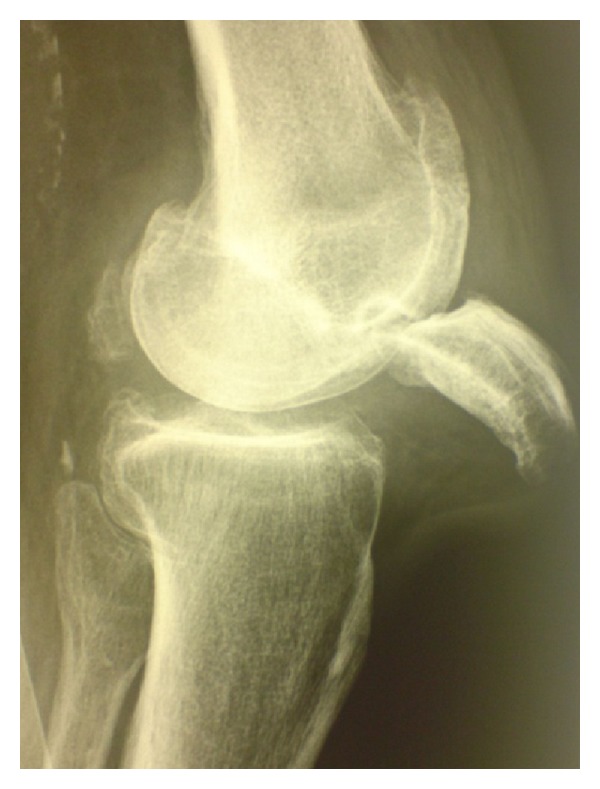
Lateral radiograph of the knee.

On examination there was an obvious deformity of the knee which was held in 40° flexion. The skin over the inferior aspect of the patella was tense (Figure 1). Any attempt at flexion resulted in patient distress. A lateral radiograph confirmed an intra-articular dislocation of the patella (Figure 2).

An unsuccessful closed reduction was made in the ED using intravenous midazolam (5 mg) and morphine (5 mg). The patient was then taken to the operating theatre where, under general anaesthesia, a further failed closed reduction and subsequent open reduction were performed.

**Figure 3:**
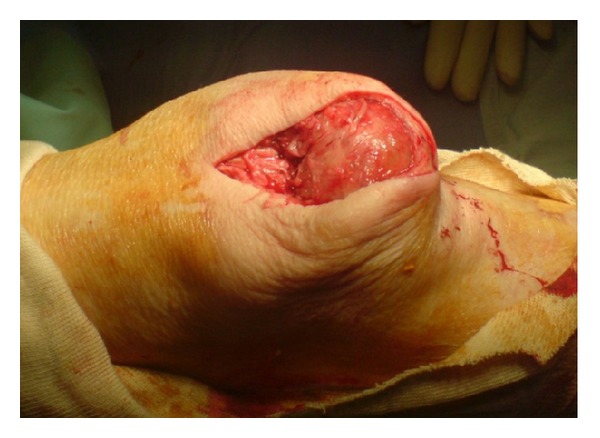
Intraoperative picture with patella dislocated.

**Figure 4:**
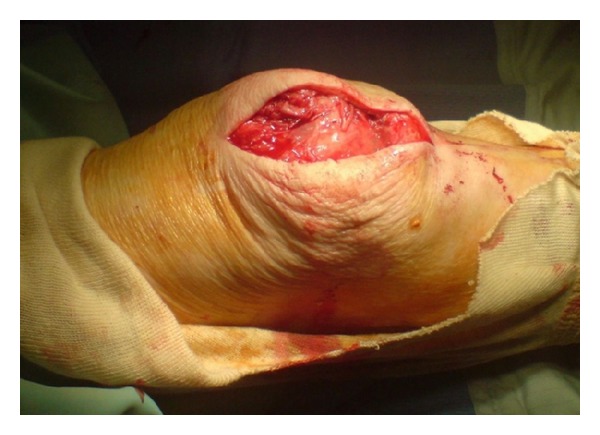
Postreduction picture.

A midline longitudinal incision was made over the patella extending proximally over the quadriceps tendon (Figure 3). Reduction at this stage was still not possible. The quadriceps tendon was opened longitudinally until the upper pole of the patella was visible. The main portion of the tendon was intact. Adhesions were then divided medially and laterally to mobilize the patella. With traction on the patella using a bone hook through the upper pole, it was then possible to reduce it (Figure 4). Postoperatively, the patient was immobilized in an extension brace for 3 weeks, and following rehabilitative physiotherapy, she returned to preinjury mobility.

## 3. Discussion

Although well described in case reports in the literature, intra-articular dislocation of the patella is an uncommon phenomenon. The dislocation can involve rotation about either the horizontal (as in this case) or vertical axis [1]. The quadriceps mechanism may rupture completely or partially in order to facilitate the dislocation [2]. The mechanism of injury is usually a direct blow onto the patella with the knee flexed. Two age groups are typically involved, adolescents and the elderly. In adolescents, it is important to be aware of the possibility of a concomitant sleeve fracture of the superior pole in association with the patellar dislocation [3].

Recent case reports of inferior dislocation have mostly involved elderly females and closed reductions were possible [4–8]. Choudhary reported a similar case in a 92-year-old lady, but following general anaesthesia, a closed reduction was possible [8]. The authors suggested that if the patellar rotation was less than 90°, then a closed reduction was appropriate. In our case, chronicity of the event may have played a part in failure to achieve a closed reduction.

The quadriceps tendon is inserted into the superior pole of the patella and is composed of 3 distinct layers. The superficial lamina is derived from rectus femoris; the intermediate lamina is derived from vastus lateralis and vastus medialis; and the deep lamina is derived from vastus intermedius. The three laminae are however firmly fused through the interlinking of their tendinous fibres. An extension of the fibres continues over the anterior surface of the patella and blends distally with the patellar ligament.

A direct blow onto the proximal pole of the patella with the knee flexed can force the patella to rotate about a horizontal axis. The deep fibres of the quadriceps tendon are avulsed allowing the patella to hinge on the intact superficial fibres. The quadriceps then prevents spontaneous reduction, and, in the elderly, superior pole osteophyte can become locked in the intercondylar notch.

It is important to maintain a high index of suspicion for these uncommon injuries. Early specialist referral is advised to follow diagnosis. Reduction is usually possible by closed manipulation either under sedation or general anaesthesia. Prognosis is good and patients should return to previous mobility levels.
